# Physiological and pathological roles of the transcriptional kinases CDK12 and CDK13 in the central nervous system

**DOI:** 10.1038/s41418-024-01413-3

**Published:** 2024-11-12

**Authors:** Consuelo Pitolli, Alberto Marini, Claudio Sette, Vittoria Pagliarini

**Affiliations:** 1 Department of Neuroscience, Section of Human Anatomy, Catholic University of the Sacred Heart, 00168 Rome, Italy; 2 GSTEP-Organoids Research Core Facility, IRCCS Fondazione Policlinico Universitario Agostino Gemelli, 00168 Rome, Italy; 3 Present Address: Saint Camillus International University of Health and Medical Sciences, 00131 Rome, Italy

**Keywords:** CNS cancer, Oncogenes

## Abstract

The cyclin-dependent kinases 12 (CDK12) and 13 (CDK13) govern several steps of gene expression, including transcription, RNA processing and translation. The main target of CDK12/13 is the serine 2 residue of the carboxy-terminal domain of RNA polymerase II (RNAPII), thus influencing the directionality, elongation rate and processivity of the enzyme. The CDK12/13-dependent regulation of RNAPII activity influences the expression of selected target genes with important functional roles in the proliferation and viability of all eukaryotic cells. Neuronal cells are particularly affected by the loss of CDK12/13, as result of the high dependency of neuronal genes on RNAPII processivity for their expression. Deregulation of CDK12/13 activity strongly affects brain physiology by influencing the stemness potential and differentiation properties of neuronal precursor cells. Moreover, mounting evidence also suggest the involvement of CDK12/13 in brain tumours. Herein, we discuss the functional role(s) of CDK12 and CDK13 in gene expression regulation and highlight similarities and differences between these highly homologous kinases, with particular attention to their impact on brain physiology and pathology. Lastly, we provide an overview of CDK12/13 inhibitors and of their efficacy in brain tumours and other neoplastic diseases.

## Facts

- CDK12 and the closely related CDK13 are members of the transcription-associated cyclin-dependent kinase family
- CDK12 and CDK13 regulate multiple layers of gene expression, including transcription, mRNA processing, and translation
- CDK12 and CDK13 play a key functional role during brain development by regulating the proliferation and differentiation of neuronal progenitor cells
- Deregulation of CDK12 and CDK13 activity is associated with human cancer
- CDK12 and CDK13 represent potential therapeutic targets for the treatment of brain tumours and other neoplastic diseases

## Open questions

- Given the low sequence homology between CDK12 and CDK13 except for the kinase domain, is it possible to imagine a functional role other than that of kinase different for each?
- Is the expression of CDK12 and CDK13 finely regulated during brain development in human?
- What are the factors that directly or indirectly make CDK12 and CDK13 oncogenes or tumour suppressors in different tumour contexts?
- Given the involvement of CDK12 and CDK13 in learning and memory mechanisms in *Drosophila Melanogaster*, do the two kinases have a functional role in the onset and progression of neurodegenerative diseases?
- Can the development of selective inhibitors for CDK12 and CDK13 increase their spectrum of applications for cancer therapy?

## Introduction

**Fig. 1:**
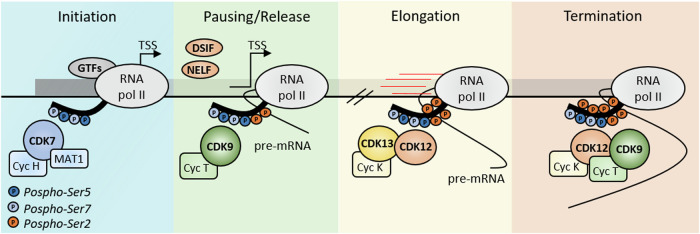
Transcription-associated CDKs-dependent phosphorylation of RNAPII CTD during the transcriptional cycle. The figure shows the different steps of transcriptional cycle and the phosphorylation of CTD RNAPII mediated by CDK7, CDK9 and CDK12/13 during the initiation, pausing/release, elongation and termination phases, respectively.

Cells of multicellular organisms need to rapidly respond to stimuli in order to maintain their homeostasis. Cellular adaptation to environmental and intrinsic cues mainly occurs through the regulation of gene expression [1]. RNA is first transcribed from the template DNA into precursor messenger RNA (pre-mRNA) by the RNA polymerase II (RNAPII) [2]. The transcription cycle consists of four main phases: initiation, promoter-proximal pausing, elongation and termination. A complex series of events dictates the transition from one phase to the other and is orderly regulated during this cycle [2]. Although an in-depth description of the molecular mechanisms underlying the regulation of the transcription cycle is beyond the scope of this review (see reviews on this issue [2, 3]), we will briefly describe how this cycle is orchestrated by the regulated phosphorylation of the carboxy-terminal domain (CTD) of the RNAPII. This highly regulated post-translational modification modulates the interaction of the RNAPII with transcriptional initiation, elongation and termination complexes, thus governing the directionality, elongation rate and processivity of the polymerase [2]. A predominant role in this regulation is played by transcription-associated (TA) cyclin-dependent kinases (CDKs), which orderly phosphorylate specific residues in the RNAPII CTD and dictate the timing and execution of the transcription cycle [4]. The RNAPII CTD consists of >50 repeats of a conserved heptapeptide: tyrosine-serine-proline-threonine-serine-proline-serine (YSPTSPS). Thus, except for the two prolines, the residues of this heptapeptide can all be potentially phosphorylated, forming an extremely flexible molecular code with great regulatory versatility [5]. Importantly, phosphorylation of Serine 2 (S2) and S5 are highly linked to transcription regulation. At the initiation phase, phosphorylation of S5 by CDK7 ensures the transition of RNAPII from the transcription start site (TSS) to the pausing site, 25-50 base pairs (bp) downstream of the TSS (Fig. 1). Then, phosphorylation of S2 by CDK9 allows pausing release and transition to the elongation phase. Phosphorylation of S2 progressively increases within the gene unit, due to the action of the highly homologous CDK12 and CDK13 enzymes, reaching a peak near the transcription end site (TES; Fig. 1). Here, phosphorylation of S2 by both CDK9 and CDK12 was proposed to couple transcript cleavage and polyadenylation with transcription termination and release of RNAPII from the template DNA [2, 4]. Thus, the fine-tuned activity of different TA-CDKs is crucial to ensure the correct progression of the transcription cycle (Fig. 1).

An additional layer of complexity in the transcriptional cycle is determined by the organization of most eukaryotic genes in multiple “coding” exons separated by long, “non-coding” introns. Therefore, the pre-mRNA needs to undergo maturation before being utilized by the cell. In addition to the capping of the 5′ end, which protects the nascent pre-mRNA from nuclease-mediated digestion, splicing of introns, cleavage of the transcript and consequent polyadenylation of the free 3′ end are all essential steps that ensure maturation and usability of the newly transcribed RNA [6]. These pre-mRNA processing events are strictly coupled with transcription, and mounting evidence indicates that TA-CDKs contribute to modulate these maturation processes [4].

Herein, we focus on the role of two TA-CDKs, CDK12 and CDK13, with particular attention to their functional relevance in the regulation of late stages of the transcription cycle (i.e. elongation and termination), co-transcriptional RNA maturation processes (i.e. splicing) and translation. The last part of this review will then focus on the molecular mechanisms through which CDK12/13 activities impact on brain physiology and pathology.

## Structural features of CDK12/13

CDK12/13 are part of the large family of CDKs, comprising 20 serine/threonine kinases that regulate multiple cellular processes. According to their main functions within the cell, CDKs can be divided in two subgroups: (*i*) cell cycle-associated CDKs (including CDK1, CDK2, CDK4 and CDK6) that regulate progression through the cell cycle; (*ii*) TA-CDKs (including CDK7, CDK8, CDK9, CDK11, CDK12 and CDK13) that are implicated in the regulation of transcription. Functionally, all these kinases share the need to bind to a cyclin regulatory subunit for their activity. However, while cell cycle-associated CDKs can bind multiple cyclins, each TA-CDK is activated by a single specific cyclin. For instance, CDK12 and CDK13 are exclusively activated by the binding to cyclin K [7]. Structurally, all CDKs have a similar two-lobed structure, with a C-terminal lobe composed of α-helices and an N- terminal lobe rich in β-sheets [7]. The C-terminal lobe contains the T-loop motif with an activating phosphorylation site. The N-terminal lobe contains the glycine-rich loop (G-loop) that harbours two inhibitory sites. Phosphorylation of the G-loop by two inhibitory kinases, WEE1 and MYT1, reduces the affinity of CDKs for their substrates, causing their functional inhibition. The CDK catalytic domain is situated between the two lobes and contains the ATP-binding site that is essential for the phosphorylation reaction [8–10].

CDK12/13 activation, as for all other CDKs, occurs in two steps, with binding of the cyclin K subunit preceding the phosphorylation of the newly assembled CDK-cyclin complex [7, 10]. In general, cyclins bind to their specific CDK by interacting with a cyclin-binding domain characterized by a highly conserved motif of 16 amino acids, named PSTAIRE sequence, located in the N-terminal lobe of the CDK. The association with the cyclin causes a conformational change of the kinase and configures its catalytic site for proper binding of ATP and of the protein substrate. Indeed, in the cyclin-free CDK, the T-loop domain masks the catalytic site preventing its activation, and it must be displaced for full activation of the enzyme to occur. Binding of the cyclin to the CDK modifies the structure and the position of the T-loop domain, thus unmasking the catalytic site and allowing substrate entry. Concomitantly, these conformational changes promote phosphorylation of T161 in the T-loop by CDK-activating kinases (CAKs), which induce additional conformational rearrangements of the CDK and improve the stability and specificity of its binding to the substrate [7, 10–14].

**Fig. 2:**
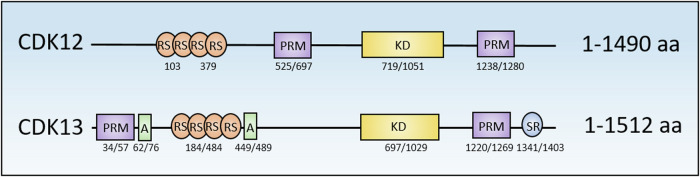
Graphic representation of CDK12 and CDK13 domains. The figure shows the comparison between protein domains belonging to CDK12 and/or CDK13. PRM proline-rich motif, A alanine-rich motif, RS arginine/serine-rich motif, KD kinase domain, SR serine-rich motif.

CDK12 (1490 amino acid-long) and the closely related CDK13 (1512 amino acid-long) are the largest members of the TA-CDK family [4] and are expressed in all tissues. CDK12/13 comprise a carboxy-terminal kinase domain, two proline-rich motifs (PRM) involved in protein–protein interactions, and an arginine/serine-rich (RS) domain, which is typical of many RNA binding proteins (RBPs) involved in splicing regulation (Fig. 2) [15–18]. Furthermore, CDK13 contains an additional serine-rich (SR) domain and two alanine-rich (A) domains, with unknown functions, that are not present in CDK12 (Fig. 2) [18]. These sequences are responsible for the low overall sequence homology (50%) between CDK12 and CDK13 [18] and may underlie the different functional activities reported for these kinases in some studies [19, 20]. Indeed, although their kinase domains are nearly identical (92%) in terms of amino acid sequence, CDK12 /13 were shown to regulate different sets of genes and to have a distinct repertoire of substrates [18, 19, 21, 22].

## Role of CDK12/13 in transcription elongation and termination

The RNAPII initially transcribes a short sequence (25–50 bp from the TSS) and then undergoes an RNA quality control mechanism known as promoter-proximal pausing. RNAPII pausing is established by the direct binding of the *negative elongation factor* (NELF) and *DRB-sensitivity inducing factor* (DSIF) to the newly assembled transcription machinery [23]. The subsequent phosphorylation of the S2, S5 and S7 residues of the CTD, as well as phosphorylation of NELF and DSIF by CDK9-cyclin T1 complex [*positive transcription elongation factor b* (P-TEFb) complex], allows the release of the paused RNAPII and the entry into the elongation phase of the transcriptional cycle [24–26]. S2 phosphorylation is also a key event to maintain a proficient elongation rate within the whole transcription unit. This modification of the CTD, which progressively augments as the RNAPII proceeds [4], is mediated by CDK12 and/or CDK13. Phosphorylation by both kinases was shown to enhance the processivity of the RNAPII (i.e. the ability to elongate through the entire length of the transcription unit), its elongation rate (i.e. the number of nucleotides incorporated in the nascent RNA transcript per unit of time) and its directionality [20, 27, 28]. The regulation of RNAPII transcriptional rate by CDK12/13 also prevents the selection of proximal intronic alternative polyadenylation (IPA) sites, thus guaranteeing the synthesis of full-length RNA transcripts and avoiding premature termination of transcription [20, 29, 30].

Recent experimental evidence showed that a proper transcription elongation is essential for survival of eukaryotic organisms [31, 32]. Accordingly, reduced elongation rate resulted in early embryonic lethality in mice [31]. Moreover, consistent with the coupling of transcription with splicing, an appropriate transcription elongation rate was also shown to regulate alternative splicing [33]. Indeed, the expression of a mutant RNAPII displaying a decelerated transcription rates in mouse embryonic stem cells (ESCs) perturbed the expression and splicing of long genes involved in synapse signalling and impaired differentiation of ESCs into neurons [31]. These observations suggest that an optimal RNAPII speed is essential for neuronal development. Moreover, conditions that de-regulate the RNAPII elongation rate, like the altered expression of CDK12/13, might particularly affect the central nervous system because of the greater length of neuronal genes [34, 35] and, therefore, their greater dependence on fast-proceeding RNAPII for expression.

There are controversial data on whether CDK12 and CDK13 act in a cooperative manner, showing functional redundancy, or, conversely, whether the activity of CDK12 is preponderant compared to that of CDK13. Earlier studies showed that while depletion of CDK12 strongly reduced RNAPII S2 phosphorylation, knockdown of CDK13 caused only partial or no effects [36, 37]. However, more recent studies have described gene-selective and non-overlapping roles for these kinases in regulating the expression of distinct set of genes, with CDK12 mainly regulating the expression of DNA damage response (DDR) genes and CDK13 primarily controlling the expression of small nuclear and nucleolar RNAs [19, 20, 27, 29, 38]. Chemical and siRNA-mediated inhibition of CDK12 expression mainly resulted in the down-regulation of DDR genes, including *breast cancer type 1* (BRCA1), *fanconi anemia complementation group I* (FANCI) and *ataxia telangiectasia and rad3-related* (ATR), which are very long genes and, due to this structural feature, could be particularly dependent on a high transcriptional rate for their expression [29, 30, 39]. Furthermore, CDK12 loss favoured the premature termination of transcription products at promoter-proximal IPA, impairing the biogenesis of full-length DDR transcripts [29, 30]. This experimental evidence supports an essential role for CDK12, but not CDK13, in guaranteeing the expression of long genes by preventing their premature termination. Interestingly, this function of CDK12 involves two substrates of its kinase activity: the CTD of RNAPII and SNRNP70. The latter is a component of the splicing-associated U1 small nuclear ribonucleoprotein (snRNP) complex. In addition to its well-known role in mediating the early stages of the splicing reaction [40, 41], U1 snRNP prevents the premature termination of RNA transcripts by masking cryptic poly(A) sites (PAS) located in introns [42, 43]. In light of this, CDK12-dependent phosphorylation of U1 snRNP could be involved in its binding to these cryptic PAS. One of the main features of long DDR genes undergoing premature termination upon CDK12 inhibition is a low U1 snRNP binding sites/PAS ratio [30].Thus, length of transcription unit ( > 25 Kb), high density of intronic PAS and low number of U1 snRNP binding sites represent structural features that contribute to dependency on CDK12 activity for expression [30]. Furthermore, CDK12 also regulates transcription termination. Indeed, this kinase is required to establish the high S2 phosphorylation levels observed at the end of the gene in proximity of PAS. In turn, this post-translational modification of the CTD favours the recruitment of components of the cleavage and polyadenylation (CPA) complex, such as *cleavage stimulation factor subunit 3* (CSTF3) and *cleavage and polyadenylation specific factor 3* (CPSF3), which ensure proper 3’-end processing [28, 44].

Interestingly, selective inhibition of CDK13 elicited a more extensive impact on the cell transcriptome and phosphoproteome than CDK12 inhibition [20]. This observation supports an independent functional role played by CDK13, which has been much less investigated than CDK12 to date. At the same time, however, the selective inhibition of CDK12 or CDK13 alone only moderately affected RNAPII transcription rate, whereas dual CDK12/13 inhibition greatly reduced both S2 phosphorylation and global RNAPII activity. Collectively, these observations suggest that CDK12 and CDK13 play both redundant/cooperative roles and unique functions in the cell [20], a hypothesis that is consistent with the lethal phenotype observed in both single knockout mice [45, 46].

### Role of CDK12/13 in splicing

Splicing is the process that removes the intronic sequences from pre-mRNA to generate a mature RNA [47]. This process is operated by a macromolecular complex, termed the spliceosome, and requires the recognition of specific regulatory sequences (splice sites) located at the exon-intron boundaries [47]. However, since the splice site sequences display low stringency, additional regulatory sequences (splicing enhancers or silencers) are required to ensure their proper recognition by the spliceosome [48, 49]. Recognition of these sequences by specific RBPs promotes the inclusion or the exclusion of an exon from the mRNA, allowing each gene to encode for multiple splice variants. This process, known as alternative splicing, represents a key step in gene expression regulation and amplifies the transcriptome and proteome complexity of eukaryotic cells [49].

CDK12/13 have been shown to play a role in the regulation of alternative splicing in various contexts. In some cases, such regulation is indirect and results from their impact on RNAPII dynamics. Indeed, changes in the elongation rate of RNAPII were shown to modulate the splicing of several exons by affecting the recruitment of splicing factors near the splice sites [31, 50–55]. In this kinetic model of splicing regulation, when RNAPII travels fast on the transcription unit, weak 3’ splice sites compete with strong 3’ splice sites of downstream exons for the same 5’ splice site, thus leading to skipping of the “weak” exon. Conversely, a slow rate of RNAPII extends the window of opportunity for recognition and usage of weak 3’ splice site by the spliceosome before strong 3′ splice sites of downstream exons are transcribed. In the absence of competition, inclusion of the variable exon is favoured, leading to expression of the corresponding splice variant [31, 53, 55, 56]. This is true for class I exons, which represent 50-80% of all exons that are sensitive to variations in the RNAPII speed. However, slow transcription elongation can also enhance the skipping of other RNAPII-sensitive exons (20–50%), termed class II exons, by causing the recruitment of splicing inhibitory factors to their splice sites [31, 53, 55, 56]. In addition, phosphorylation of the RNAPII CTD at the S2 residue was also shown to directly affect splicing decisions by recruiting constitutive splicing factors, such as U2AF65 [57].

CDK12 was shown to regulate the selection of alternative last exons (ALEs) in long genes, such as DDR genes, prevalently by favouring the usage of distal ALEs and the expression of the longer splice variants [58]. The regulation of ALEs by CDK12 is highly gene- and cell-specific, and may partly involve the CDK12-dependent recruitment of gene-specific polyadenylation factors on transcription termination motifs in the 3’ UTRs of the regulated ALEs [58]. The activity of CDK12/13 is required to globally suppress IPA events, enabling the production of full-length gene products [20, 29, 30, 59]. Therefore, these kinases could be required to maintain the transcriptional machinery in an active elongating status to prevent premature transcription termination and to ensure the proper splicing of the downstream exons. At the same time, it is also possible that CDK12/13 regulate splicing independently of S2 phosphorylation of the RNAPII CTD. Recently, a phosphoproteomic screening identified 26 candidate CDK12 substrate proteins involved in transcriptional regulation, RNA processing and chromatin modification [60]. Among them, an interesting protein is LEO1, a subunit of the *polymerase-associated factor 1 complex* (PAF1C). Depletion of LEO1, or expression of a LEO1 kinase-dead mutant, attenuated the association of PAF1C with the RNAPII and impaired transcription elongation [60]. This study highlights the concept that CDK12 may also affect the transcriptional rate of RNAPII by regulating the activity of co-factors that are essential to guarantee a proper elongation rate.

**Fig. 3:**
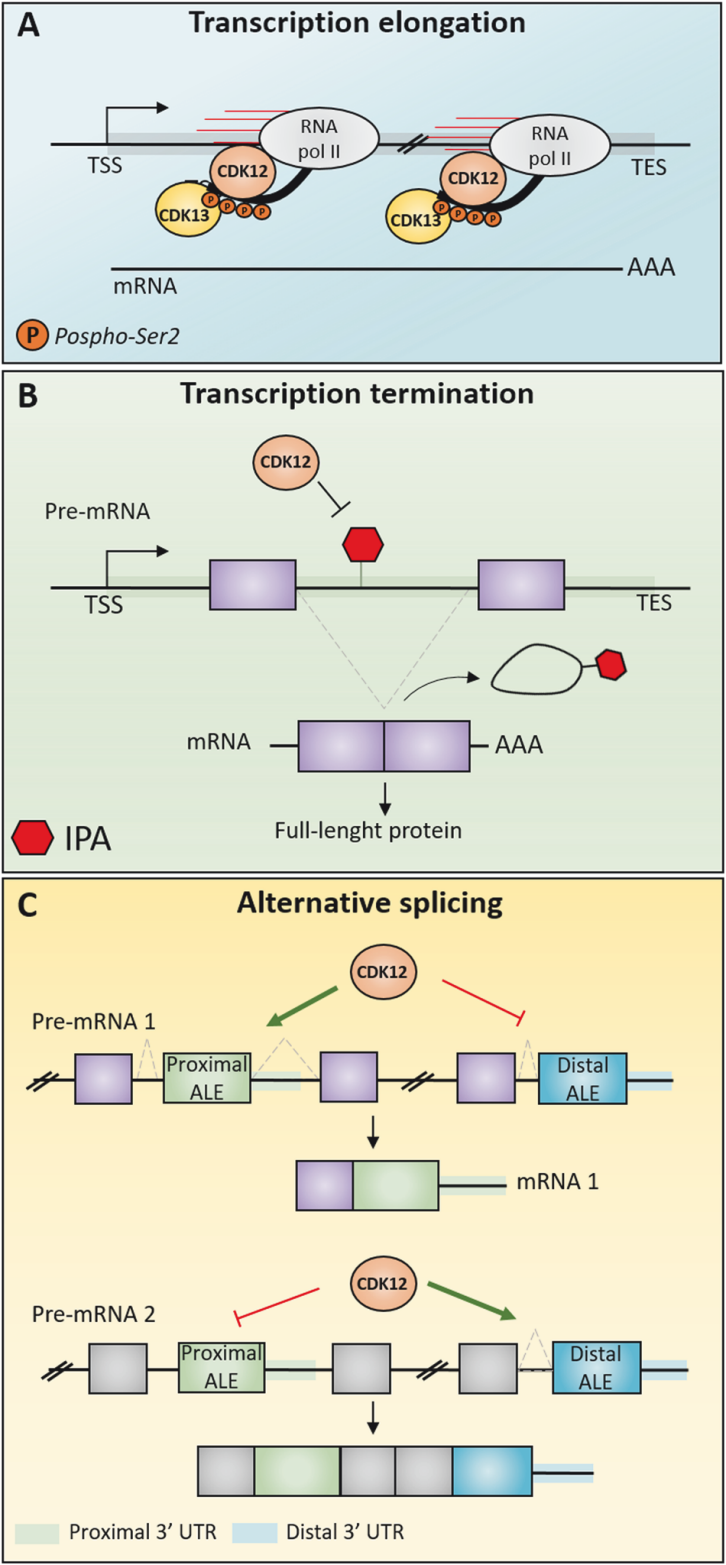
Role of CDK12/13 in regulating gene expression. **A** CDK12/13 regulate the elongation phase of transcription by phosphorylating RNAPII CTD at S2 residue. **B** CDK12 prevents pre-mRNA premature termination by impairing the use of promoter-proximal IPA. **C** CDK12 modulates alternative last exon (ALE) splicing by favoring the expression of several mRNA transcript variants with different 3′ ends.

Splicing regulation by CDK12/13 may also rely on their ability to phosphorylate splicing factors or, more in general, RBPs. These post-translational modifications likely regulate RBP expression, localization and activity [20, 27, 58, 61]. Interestingly, both CDK12 and CDK13 contain multiple RS domains, which are commonly present in splicing factors, and localize within nuclear speckles [16, 17], the well-characterized nuclear structures enriched in components of the spliceosome. Moreover, CDK12/13 regulate the interaction between S2 phosphorylated RNAPII and SF3B1, the protein component of the U2snRNP that binds to the branchpoint at the 3’ splice site [62]. CDK12/13 inhibition disrupted this interaction and reduced the recruitment of SF3B1 to the weak 3’ splice site of introns that require CDK12/13 activity for efficient splicing [62]. This study also showed that inhibition of CDK12/13 allows transcription to proceed beyond the promoter-proximal introns that are regulated, but it selectively impairs their splicing [62]. Thus, retention of these introns was not the direct consequence of transcription inhibition, but rather a splicing defect caused by impaired recruitment of SF3B1 to weak splice sites. In this scenario, IPA of some CDK12/13 target transcripts may represent the consequence of prolonged intron retention due to inefficient splicing, highlighting the key role played by these kinases in the regulation of transcription-coupled RNA processing events (Fig. 3).

### Role of CDK12/13 in translation

Cap-dependent translation of eukaryotic mRNAs starts with the binding of the multiprotein *eukaryotic initiation factor* (eIF) 4 F complex to the 7-methyl-GTP cap, a structure present at the 5′ terminus of the transcripts [63]. EIF4F is composed by the cap-binding protein eIF4E, the helicase eIF4A and its cofactor eIF4B, and the scaffold protein eIF4G. This complex binds to the mRNA and allows the recruitment of ribosomes near the 5′ terminus, promoting codon scanning until the ribosome reaches the first AUG and translation begins [63]. Under physiological conditions, the function of eIF4E is tightly regulated by the binding of inhibitory proteins, termed eIF4E binding proteins (4E-BPs), which prevent its association with eIF4G and inhibit translation initiation [63]. Upon mitogenic stimulation, 4E-BPs are phosphorylated by the *mechanistic target of rapamycin* (mTOR) kinase, causing its dissociation from eIF4E, assembly of the eIF4F complex and initiation of translation [63].

Experimental evidence of the involvement of CDK12/13 in the regulation of translation was first gathered in *Saccharomyces cerevisiae* [64–66]. The yeast ortholog of CDK12/13, Ctk1, was implicated in both translation initiation and elongation by, respectively, promoting the formation of mature 80S ribosomes [66] and regulating the phosphorylation of the small 40S subunit component of Rps2 [64, 67]. These observations highlighted a key role for the yeast Ctk1 to maintain high decoding fidelity during protein synthesis [64, 66, 67]. A direct involvement of mammalian CDK12 in the regulation of translation was also reported. CDK12 directly phosphorylates 4E-BP1 at S65 and T70 residues, thus favouring the exchange of 4E-BP1 with eIF4G at the 5′ cap and efficient translation of many mRNAs that are target of the mTOR complex 1 (mTORC1) [68]. This experimental evidence was further confirmed by a study that uncovered a synergism between EGFR (Gefitinib) and CDK12/13 (THZ531) inhibitors in inducing cytotoxicity in triple negative breast cancer (TNBC) [69]. Chemical inhibition of CDK12/13 strongly reduced the phosphorylation of 4E-BP1 and this effect was enhanced by combined treatment with Gefitinib [69]. The combined treatment strongly impaired the translation of oncogenic proteins, such as MYC and MCL-1, and increased their proteasome-dependent degradation, thus favouring tumour cell death [69]. However, since CDK12 is predominantly localized in the nucleus [16, 70], it remains to be elucidated the molecular mechanism/s by which CDK12 phosphorylates a regulatory factor of translation initiation like 4E-BP1 that is prevalently localized in the cytoplasm. An interesting possibility is that the assembly of the translation initiation complex might co-transcriptionally occur on nascent transcripts in the nucleus [61]. Thus, CDK12 might also be involved in the export of mRNAs from the nucleus to the cytoplasm where they will be promptly translated, a role that has been shown for other TA-CDKs, like CDK9 [71].

Unlike the global effect of mRNA translation initiation and elongation exerted by the yeast Ctk1 [64, 66], CDK12 activity appears to control translation of a limited (∼1000) number of mRNAs that are enriched in functional categories related to DNA repair, RNA processing, cell proliferation, mitochondria function and mitosis [68]. Among them, several proteins are involved in the centromere-kinetochore architecture, mitotic spindle regulation and chromosome segregation (i.e. subunits of the CENP, CEP, and SMC complexes as well as NDC80, NUF2, MIS12) [68]. Accordingly, CDK12 depletion resulted in severe chromosome misalignments, defective spindle-kinetochore attachments and a dramatic increase in the metaphase-arrested cells [68]. Thus, it is possible that the defective DNA repair and the profound genomic instability observed in metastatic cancers harbouring CDK12 loss-of-function mutations [72–74] are in part also due to the translation defects of mitosis-related transcripts. Depending on the tumour context, CDK12 might contribute to the oncogenic phenotype by favouring the translation of oncogenic proteins [69], while guaranteeing genome stability by ensuring the proper translation of mitosis-related proteins [68].

Another link between CDK12/13 and translation regulation is represented by the regulation of the expression of RAPTOR, a positive regulator of mTORC1 [75]. Pharmacologic inhibition of both kinases by treatment of ovarian cancer cells with THZ531 caused the up-regulation of a proximal ALE event in intron 10 of RAPTOR, leading to the downregulation of more distal exons and repression of RAPTOR protein expression [59]. Coherently, selection of the ALE event in RAPTOR correlated with reduced phosphorylation of the mTORC1 substrate 4E-BP1 [59], indicating another mechanism through which CDK12/13 impact on translation regulation.

Similar connections between these CDKs and translation were also reported in colorectal cancer cells. In particular, CDK13 was shown to directly interact and phosphorylate eIF4B and 4E-BP1, leading to increased protein synthesis efficiency [76]. As also shown in TNBC cells [69], CDK13 positively regulates the translation of MYC [76], which is functionally relevant for the viability and proliferation of colorectal cancer cells downstream of CDK13 [76]. Thus, it is likely that CDK12/13 coordinate translation initiation by directly or indirectly catalysing phosphorylation of multiple components of the translational machinery and controlling the expression of pro-tumoral players, like MYC or MCL-1, that are essential for tumour progression and acquisition of chemoresistance by cancer cells.

## CDK12/13 in brain development

CDK12/13 are widely expressed in the different anatomic structures (i.e. forebrain, midbrain, primitive streak and tail bud mesoderm) that are gradually formed during brain development [77]. In the adult brain, cerebellum expresses the highest levels of these kinases [78]. These observations argue in favour of their functional relevance for the execution of both developmental and adult brain programs. The first experimental evidence which highlighted the functional relevance of CDK12 during development was obtained in murine ESCs, cells derived from the inner cell mass (ICM) at the blastocyst stage [45]. Murine ICM cells proliferate rapidly between embryonic day 3.5 (E3.5) and E6.5 and then differentiate into the three germ layers of the developing embryo. Knockout of the *Cdk12* gene in mice (*Cdk12*^*KO*^) caused a lethal phenotype during the peri-implantation stage, with no embryos surviving beyond E6.5 [45]. Mechanistically, CDK12 depletion strongly reduced the expression of DDR genes and triggered accumulation of double strand breaks (DSBs) [45]. This high level of DNA damage exceeds the repair capacity of CDK12-depleted ICM cells, preventing their proliferation and inducing cell senescence, apoptosis and death of the embryo [45].

**Fig. 4:**
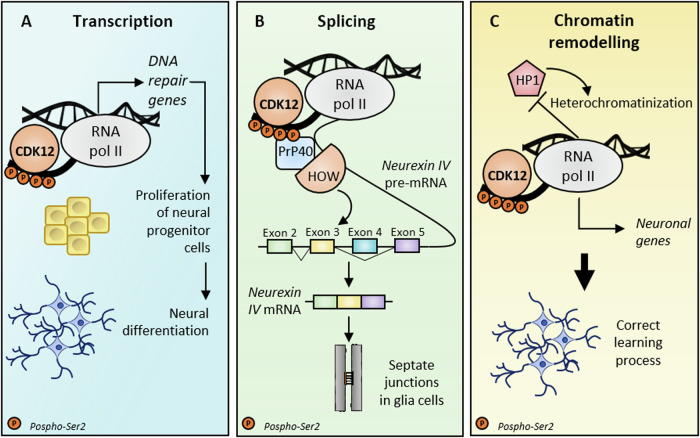
CDK12 is involved in neuronal development by regulating transcription, splicing, and chromosomal packaging. **A** During embryonal neurogenesis in mice, CDK12 fosters the proliferation of NPCs in neural tube by promoting the transcription of genes involved in the DNA repair process. Conditional deletion of CDK12 in NPCs leads to microcephaly, petite cerebral cortex, and aberrant corpus callosum, resulting in death after birth (P1) [79]. **B** By ensuring a correct splicing program of the septate junction cell adhesion molecule Neurexin IV pre-mRNA, CDK12 allows the formation of a functional BBB. In flies, CDK12 S2 phosphorylation of the RNAPII CTD favors the recruitment of the complex Prp40/HOW, which in turn engages the spliceosome on Neurexin IV pre-mRNA and promotes the inclusion of exon 3 and the exclusion of exon 4 in the mature mRNA [86]. **C** The learning process in *Drosophila Melanogaster* is severely impaired by CDK12 depletion. In normal conditions, CDK12-dependent S2 phosphorylation of the RNAPII CTD maintains the process of transcription elongation, preventing the ectopic accumulations of heterochromatin and promoting the expression of genes involved in regulating learning acquisition. Conversely, the heterochromatinization caused by CDK12 loss induces the transcription repression of such genes, thus impairing neuronal functions and behaviors [84].

Conditional depletion of *Cdk12* in the mouse neural tube starting from E10.5 postponed the death of the mice until few hours after birth at postnatal day 1 (P1). This study also highlighted the essential role played by CDK12 in regulating cerebral cortex development (or corticogenesis) by controlling the proliferation of neural progenitor cells (NPCs) [79]. Exit from the cell cycle and differentiation of NPCs is essential for the establishment of the pool of neurons that populate the developing cortical plate. CDK12 controls the proliferation and the viability of NPCs by ensuring an adequate expression of DDR genes [79]. Accordingly, ablation of *Cdk12* in the neural tube disrupted neurogenesis and caused a reduction in size of the cerebral cortex and microcephaly [79]. Furthermore, other portions of the central nervous system were also affected by CDK12 depletion. The olfactory bulb and cerebellum were smaller in *Cdk12*^*KO*^ mice compared to control mice, while histological analysis of brains at birth exhibited an abnormal corpus callosum, with axons that failed to cross into the contralateral cerebral hemisphere [79]. In addition, several axonal tracts (i.e. anterior commissure, internal capsule, posterior commissure) showed morphological alterations and were profoundly reduced in size [79], suggesting that CDK12 can regulate the expression of not yet identified targets that control other critical steps associated with brain development or with functions of the mature organ (Fig. 4).

CDK12/13 have also been shown to control axonal extension [77] and the migration of late-arising neurons (E15.5) to the cortical plate during corticogenesis [79]. Interestingly, both these regulations are mediated by the transcriptional down-regulation of *Cdk5* [77, 79], a member of the CDKs family highly expressed in post-mitotic neurons and known to phosphorylate cytoskeletal proteins, thus affecting the maturation and migration of mature neurons [80–82]. Coherently, ectopic expression of CDK5 partially restored both axonal extension in differentiated P19 mouse embryonal carcinoma cells [79] and neuronal migration defect in *Cdk12* mutant mice [77, 79].

Recently, a novel essential function of CDK12 has been discovered also in adult neurons of *Drosophila Melanogaster* [83]. In this context, CDK12 controls actin polymerization in the soma-axon boundaries of adult neurons by ensuring the expression of genes associated with the catabolism of homocysteine [83]. CDK12^*KO*^ neurons displayed a specific swelling of the proximal axon region, with the formation of large axonal blebs associated with actin patches. This phenotype correlated with neuronal degeneration [83]. However, the authors did not investigate the possibility that CDK12 could regulate the actin organization in adult neurons through CDK5-dependent regulatory mechanism/s, as the studies in embryonic neurons might suggest [77, 79]. Interestingly, CDK12 loss in adult neurons correlates with altered mitochondrial dynamics resulting in an increase of fragmented mitochondria in axonal swellings. However, whether this defect represents an independent event or a consequence of actin cytoskeleton remodelling was not furtherly investigated [83]. Thus, CDK12 might represent a novel determinant for energy capacity and organelle homeostasis in the axon of adult neurons, while mitochondrial fragmentation and actin disorganization might contribute to neuronal dysfunction and neurodegeneration observed upon CDK12 depletion.

Another intriguing observation links CDK12 with learning in flies. In this model, the phosphorylation of the RNAPII CTD provides a platform for counteracting heterochromatin enrichment, possibly by modulating the recruitment of selected epigenetic factors and inducing the transcription of learning-related genes [84]. Depletion of CDK12 induced heterochromatinization in third-instar larvae, primarily on the X chromosome in proximity of highly expressed longer genes characterized by high exon number [84]. Neuronal genes encoding voltage-gated and mechanosensitive ion channels, ligand-gated channels/receptors, and other genes involved in learning were among the most affected categories undergoing transcriptional repression as a result of CDK12 loss [84]. Coherently, CDK12 ablation severely impaired the learning process in *Drosophila Melanogaster* [84] (Fig. 4). Nevertheless, it remains to be clarified whether the role of CDK12 in modulation of heterochromatinization is conserved in vertebrates and whether this kinase is involved in learning and memory acquisition also in humans. In support of this notion, CDK12 was shown to specifically regulate the expression of long genes also in mammalian cells [29, 30], and this structural feature is a common trait shared by many mammalian synaptic genes [31, 34]. Of note, heterozygous mutations within the kinase domain of CDK13 are linked with a syndromic form of intellectual disability [85], suggesting that this kinase is also involved in cognitive functions in higher eukaryotes.

In addition to directly impact on the expression of selective gene clusters that are essential for the viability and functionality of neurons, CDK12/13 may also control their splicing by influencing the recruitment of RBPs and/or splicing factors on the pre-mRNA. For instance, an RNAi-based screening in *Drosophila Melanogaster* identified CDK12 as the main kinase required for the splicing of the cell adhesion molecule Neurexin IV (Nrx-IV) [86]. Nrx-IV exons 3 and 4 are spliced in a mutually exclusive manner and give rise to two different isoforms with distinct adhesive properties. Exon 3-containing Nrx-IV transcript is highly expressed in glia cells and has a fundamental role in the generation of septate junctions and in the formation of the blood-brain barrier (BBB) [86]. CDK12-dependent phosphorylation of the RNAPII CTD recruits the spliceosome core component *Pre-mRNA processing factor 40 homolog A* (PRP40) and the STAR-family member *Held Out wings* (HOW) which, in turn, bind specific intronic sequences on the Nrx-IV pre-mRNA and promote the inclusion of exon 3 [86]. Thus, albeit indirectly, the activity of CDK12 ensures a splicing event that guarantees proper glial differentiation and the formation of the BBB (Fig. 4) [86].

Collectively, these experimental observations suggest the possibility that alterations in the function of CDK12, and possibly CDK13, are implicated in axonopathies and neurodegenerative diseases, such as Alzheimer’s disease, for which the contribution of mechanisms regulated by these kinases (i.e., modulation of CDK5 expression [87–89], alterations of actin cytoskeleton remodelling [90] and heterochromatin status [87]) to the pathogenesis have been demonstrated. Notably, the onset of neurodegenerative diseases is related to aging and recent findings have suggested age-related changes in transcriptional efficiency in a wide range of eukaryotes. In one case, it was proposed that accumulation of unrepaired DNA lesions during aging leads to global RNAPII stalling and reduced transcription elongation [91]. Such age-related transcriptional stalling occurs in a gene-length dependent manner [91]. Since *CDK12* (73.06 Kb) and *CDK13* (149.32 Kb) are considerably long genes, one could speculate that their expression is also reduced during aging. Moreover, given their role in promoting transcription elongation and the expression of DDR genes, the potential reduction in CDK12/13 expression could exacerbate this age-related phenotype. However, an acceleration of the RNAPII speed during aging was also reported in a different study [92]. In this case, aging was shown to be associated with a more open chromatin structure that allows the acceleration of RNAPII. CDK12 was previously shown to limit heterochromatinization [84]. Thus, its increased expression would be expected in this context. Nevertheless, since the two studies employed different experimental models and approaches, it is difficult to reconcile these apparently conflicting observations. Moreover, since neither study focused on brain, a direct evaluation of the role and impact of altered CDK12/13 function in brain aging and/or neurodegenerative diseases would be extremely interesting.

## CDK12/13 in brain cancers

**Fig. 5:**
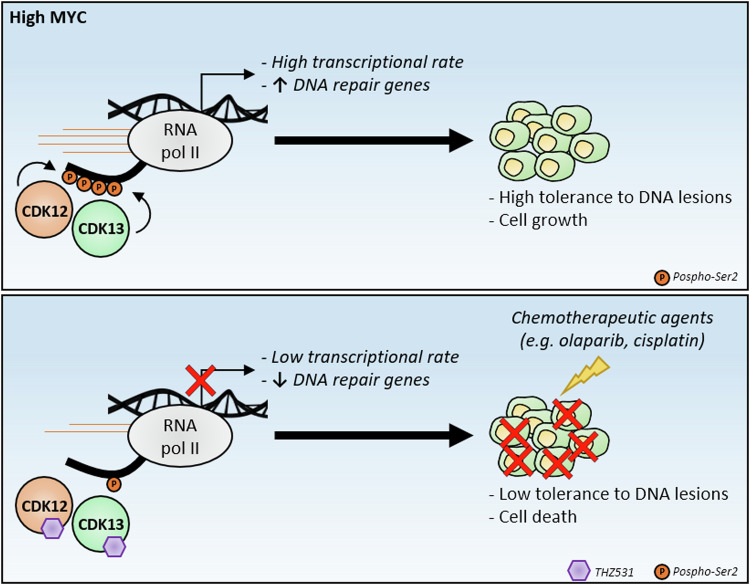
Inhibition of CDK12/13 sensitizes tumour cells to DNA damage induction. Transcriptional activated CDK12/13 guarantee a proper RNAPII elongation rate in a context of MYC-dependent high transcriptional burden, allowing the expression of DDR genes, which in turn ensure tolerance to DNA lesions (**upper panel** [94]). Simultaneous inhibition of CDK12/13 by THZ531 in Group 3 medulloblastoma cells reduces the transcription of DDR genes, leading to increased sensitivity to DNA damage inducing agents and resulting in cell death (**lower panel** [39]).

Three common and fundamental hallmarks of cancer [93], such as genome instability, epigenetic reprogramming and cancer stem cell self-renewal, are cellular processes in which the involvement of CDK12/13 has been demonstrated. As mentioned above, CDK12/13 were shown to play important functions in controlling NPCs proliferation [79], expression of DDR genes [79] and chromatin remodelling [84]. These observations strongly suggest a role of these TA-CDKs in brain tumours, although it remains unclear whether they act as tumour suppressors or oncogenes in different contexts. In this regard, it was demonstrated that the TA-CDK inhibitors THZ1, which simultaneously inhibits CDK7, CDK12 and CDK13, and THZ531, which selectively targets CDK12/13, strongly impaired viability of medulloblastoma (MB) cells, with a significant selectivity toward MYC-amplified cell lines [39, 94]. Both inhibitors reduced the expression of DDR genes and impaired DNA repair through the homologous recombination pathway, which resulted in enhanced sensitivity of MB cells to ionizing radiation [94] and DNA damaging drugs [39] (Fig. 5). MYC-dependent transcriptional amplification imposes a stress to the cell, by promoting collisions between the transcription and replication machineries, thus increasing stalling of the replication fork and DNA lesions [59, 95]. This defect might be particularly true for the MYC-amplified MB, which concomitantly displays high endogenous DNA damage and high expression of DDR genes, probably as a compensatory mechanism to guarantee cell fitness and to avoid cell death [39]. Thus, if on one hand the up-regulation of the DDR pathway is a key feature of MYC-amplified MB cells that confer dependency on CDK12/13, on the other hand it also generates an actionable vulnerability that can be exploited therapeutically [39, 94]. Nevertheless, additional studies are required to better dissect the contribution of CDK12/13 in different aspects of the physiopathology of human nervous system diseases and to pave the ground for pre-clinical and clinical studies aimed at counteracting CDK12/13 activity as therapeutic tool for brain tumours.

## CDK12/13 as therapeutic targets in human cancer

The role of CDK12/13 as oncogenes in a MYC-overexpressed context has been highlighted in other cancer models. For instance, ovarian cancers that simultaneously express high levels of MYC, CDK12 and CDK13 define a cohort of patients with a pronounced reduction of the overall survival [59]. Moreover, *CDK12* locus amplification occurs in 80 ~ 90% of *HER2*-amplified breast cancers and is associated with oncogenic features. Indeed, overexpression of CDK12 in these tumours fuels the WNT and ErbB-PI3K signalling pathways, promoting the self-renewal of cancer stem cells and tumour progression [68]. On the other hand, inactivating mutations in the *CDK12* or *CDK13* loci have also been associated with tumorigenesis. For instance, loss of CDK12 function occurs in 3–4% of patients with ovarian cancer and cause tandem duplications (TDs) that result in genomic instability [72, 96, 97]. Furthermore, ovarian cancer patients harbouring deletions of either CDK12 or CDK13 also carry mutations in at least one other DDR gene, supporting the idea that loss of CDK12/13 is associated with higher tumour mutation burden [59]. Likewise, TDs and chromosome instability were also found in 6–7% of metastatic castration-resistant prostate cancer (CRPC) harbouring bi-allelic inactivation of CDK12 [73]. Interestingly, CDK12 ablation in CRPC contributes to the progression and the aggressive phenotype of the tumour by reprogramming the energy metabolism in favour of greater cell viability and drug resistance [98].

Fewer studies have correlated CDK13 activity with cancer to date. An interesting work recently demonstrated that CDK13 promotes proliferation and invasion of thyroid cancer cells [99]. The pro-oncogenic role of CDK13 was also suggested in hepatocellular carcinomas, where the *CDK13* locus is amplified and correlates with age of clinical onset in a subset of patients [100]. However, recent evidence showed that loss of function mutations in CDK13 are found in melanoma patients, highlighting an unexpected and hitherto unproven role for CDK13 as tumour suppressor [101]. This evidence represents only a small part of the abundant literature that attests the involvement of both CDK12/13 in different types of tumours, but well highlights the dual and sometimes opposite role played by them as oncogenes or tumour suppressors depending on the tumour context. Thus, since the role of CDK12/13 in cancer is outside the scope of this review, we refer to excellent reviews on the topic [4, 102, 103].

The key functions of CDK12/13 in promoting transcription and splicing of DDR genes, together with studies reporting their oncogenic roles, make these kinases an ideal target for novel anti-cancer therapies. Interestingly, inhibition of other TA-CDKs involved in the phosphorylation of the RNAPII CTD has a significant impact on different cancer types. The CDK7 covalent inhibitor SY-1365 was shown to downregulate the expression of DDR genes, with consequent cytotoxic and cytostatic effects in several tumour types [104]. Of note, SY-1365 is now in phase I of clinical trial for advanced breast and ovarian cancer [NCT03134638, https://clinicaltrials.gov/ct2/home]. Likewise, the CDK9 inhibitors A-1592668 and AZD4573 were shown to synergize with the BCL2 inhibitor Venetoclax in haematologic tumours [105, 106]. Notably, Venetoclax is currently being evaluated in clinical trial for the treatment of advanced haematological malignancies [NCT03263637, https://clinicaltrials.gov/ct2/home].

To date, preclinical studies have highlighted the efficacy of THZ1 and THZ531, which are potent CDK12/13 inhibitors, against several types of tumours [4]. Both inhibitors were shown to downregulate the expression of DDR genes and to synergize with the PARP inhibitor Olaparib in Ewing sarcoma cells [107]. Likewise, the same mechanism involving the downregulation of DDR genes is responsible for the increased sensitivity to radiation and chemotherapeutic agents of Group 3 MB cells treated with, respectively, THZ1 or THZ531 [39, 94]. Inhibition of CDK12/13 by treatment of ovarian cancer cells and organoids with THZ531 impaired the splicing and expression also of other genes with strong relevance for tumorigenesis, such as the *epidermal growth factor receptor* (*EGFR*) and *regulatory associated protein of MTOR complex 1* (*RPTOR*) genes [59]. This effect was functionally relevant, as it enhanced the sensitivity of these ovarian cancer models to Lapatinib (EGFR inhibitor) and Everolimus (MTORC1 inhibitors) [59]. These data prompted an observational study that employs patient-derived organoids generated from high grade serous ovarian cancer samples to test the anti-tumoral efficacy of THZ531 administered alone or in combination with chemotherapeutics in use for this cancer [NCT04555473, https://clinicaltrials.gov/ct2/home].

THZ531 was also shown to improve the efficacy of spliceosome inhibitors, which displayed adverse toxic effects in clinical trials [108, 109]. The opportunity to lower the dose of splicing inhibitors by the concomitant use of CDK12/13 inhibitors may improve their clinical use by limiting toxicity. For instance, THZ531 synergized with the SF3B1 splicing factor inhibitor pladienolide B in pancreatic cancer cells [62]. Mechanistically, this effect was associated with disruption of the interaction of RNAPII with SF3B1 and consequent impairment of splicing of proximal introns in tumour-related genes.

However, despite the potent efficacy demonstrated by CDK12/13 inhibitors in preclinical studies, resistance to these agents is likely to develop in vivo. Upregulation of multidrug transporters belonging to the ATP-binding cassette (ABC) family represents the major mechanism of the acquired resistance to THZ series of covalent CDK7/12/13 inhibitors [110]. These transporters mediate drug efflux, thereby interfering with the cellular accessibility of the compound to its target [110]. The recent generation of a CDK inhibitor that is not a substrate for ABC transporters, may allow to overcome this obstacle [110], highlighting the importance of considering this common mode of resistance in the development of clinical analogs of CDK12/13 inhibitors.

## Concluding remarks

The promising anti-cancer effects of THZ1 and THZ531 in several tumours has paved the ground for the developing novel CDK12/13 inhibitors. The selective non-covalent dual inhibitor of CDK12/13 SR-4835 was recently tested in TNBC cells [111]. In this model, SR-4835 also induced IPA of DDR genes, reducing their expression and generating a “BRCAness” phenotype that, in turn, promoted a greater sensitivity to DNA damage-inducing chemotherapeutics [111]. The molecule BSJ-01-175 is a novel potent CDK12/13 covalent inhibitor, showing high capacity in reducing RNAPII S2 phosphorylation, expression of *BRCA1* and *BRCA2* genes, and tumour growth of patient-derived xenograft of TC71 Ewing sarcoma cells [112]. Furthermore, a high throughput screening study of 3-benzyl-1-(trans-4-((5-cyanopyridin-2-yl)amino)cyclohexyl)-1-arylurea derivatives has identified a promising compound with specificity for CDK12/13 [113]. Such compound inhibits RNAPII S2 phosphorylation and proliferation of SK-BR-3 breast cancer cells [113]. Lastly, Niu et al. developed the selective CDK12-CyclinK complex inhibitor PP-C8, which exploited the PROteolysis-TArgeting Chimera (PROTAC) approach by linking a target protein ligand with an E3 ligase recruiting moiety, ultimately targeting the CDK12-CyclinK complex to proteasome degradation [114]. In turn, treatment with PP-C8 resulted in DDR gene downregulation and synthetic lethality in combination with Olaparib in MDA-MB-231 triple-negative breast cancer cell line [114].

Comparing to the first generation of pan-CDKs inhibitors, such as Dinaciclib, which targeted multiple CDKs with similar affinities [115], the abovementioned more selective molecules showed no obvious adverse effects in non-tumoral context. For instance, THZ1 treatment was shown to cause no adverse effects in non-tumour-bearing mice [116]. However, it is important to highlight that THZ1 is able to suppress RNAP II CTD phosphorylation both in tumour and normal cells, albeit the pro-apoptotic effect was only observed in cancer cells [117]. Regarding THZ531, although no adverse effects in normal cells have been reported so far, its therapeutic use is limited by low solubility and poor metabolic stability [112]. These limitations have been overcome by its derivative compound BSJ-01-175 [112]. This is the first dual CDK12/13 inhibitor showing an in vivo efficacy in mice, although a slight weight loss was reported and a reconsideration of the tolerable dosage is required [112]. Importantly, the non-covalent CDK12/13 inhibitor SR-4835 elicited only a transient induction of DNA damage in normal cells and no toxic effects were observed in vivo [111]. These observations suggest that the new generation of CDK12/13 inhibitors, which target transcription and DDR, are very specific to tumour cells addicted to these biological processes. Furthermore, an accurate design of dosing, together with the potential use in combination with other drugs (such as splicing inhibitors and genotoxic drugs), may further reduce the possible adverse effects. In light of this, the recent development of CDK12/13-selective degraders [114] represent an excellent strategy to improve drug specificity and minimize undesired effects.

In conclusion, given the widespread impact of CDK12/13 on gene expression at different layers of regulation and the high anti-tumoral efficacy of CDK12/13 inhibitors alone or in combination with other chemotherapeutic agents, it is hoped in the near future an increased number of clinical trials especially for brain tumour patients for whom very few therapeutic options are currently available.
